# Smear positive extra pulmonary tuberculosis disease at University of Gondar Hospital, Northwest Ethiopia

**DOI:** 10.1186/1756-0500-6-21

**Published:** 2013-01-18

**Authors:** Yohannes Zenebe, Belay Anagaw, Wogahta Tesfay, Tewodros Debebe, Baye Gelaw

**Affiliations:** 1 Department of Microbiology, Immunology and Parasitology, College of Medicine and Health Sciences, Bahir Dar University, Bahir Dar, Ethiopia; 2 Department of Medical Microbiology, School of Biomedical and Laboratory Sciences; College of Medicine and Health Sciences, University of Gondar, Gondar, Ethiopia; 3 Department of Pathology, School of Medicine; College of Medicine and Health Sciences, University of Gondar, Gondar, Ethiopia

**Keywords:** Extra pulmonary tuberculosis, Acid fast bacilli, Northwest Ethiopia

## Abstract

**Background:**

While pulmonary tuberculosis is the most common presentation, extra pulmonary tuberculosis is also an important clinical problem. However, no adequate information had been made available on the prevalence of smear positive extra pulmonary tuberculosis in Gondar. The aim of this study was to assess the prevalence and possible risk factors of smear positive extra pulmonary tuberculosis among suspected patients at University of Gondar Hospital.

**Methods:**

A cross-sectional study on extra pulmonary tuberculosis suspected patients was conducted at University of Gondar Hospital from January 2012 to April, 2012. Specimens of patients suspected of extra pulmonary tuberculosis were obtained from fine needle aspiration and body fluid samples collected by pathologist. Demographic characteristics and other variables were collected using a pretested semi-structured questionnaire. Smears were prepared from each sample and stained by Ziehel Neelson and Wright stain. The result of the study was analyzed with bivariate and multivariate logistic regression.

**Result:**

A total of 344 extra pulmonary tuberculosis suspected clients were included in the study and specimens were taken from lymph node aspirates and body fluids. The overall prevalence of smear positive extra pulmonary tuberculosis was 34 (9.9%). Of these cases of extra pulmonary tuberculosis, lymph node tuberculosis constituted the largest proportion (82.4%). Among the 34 extra pulmonary tuberculosis patients, over half of them (52.9%) were positive for human immunodeficiency virus. The largest proportion of tuberculosis and human immunodeficiency virus cases occurred among persons with in the age group of 31–40 years. Previous history of tuberculosis (OR = 4.77, 95% CI 1.86-12.24), contact to a known tuberculosis cases (OR = 6.67 95% CI 2.78-16.90), history of underlying diseases (OR = 2.79 95% CI 1.15-6.78) and income (OR = 12.9 95% CI 2.25-68.02) were significantly associated with extra pulmonary tuberculosis infection.

**Conclusion:**

The prevalence of smear positive extra pulmonary tuberculosis infection in Gondar is high. Screening of lymph node and other body fluid specimens for extra pulmonary tuberculosis could help for treatment, control and prevention of the disease.

## Background

Tuberculosis (TB) is one of the leading causes of death in the world. Globally around 8.8 million people develop tuberculosis and 1.45 million people die every year due to TB [1]. An increased incidence of tuberculosis occurs mostly in Africa and Asia, where the highest prevalence of co-infection with HIV and *M*. *tuberculosis* occur [2,3]. According to the World Health Organization (WHO) 2007 report, around 31% of TB cases were in Sub-Saharan Africa and 14.8% of these being among people living with human immunodeficiency virus (PLWHIV) [4]. The global burden of death and disease caused by TB is concentrated particularly in low-income countries. Sub-Saharan Africa is the high prevalent area in TB infection .The WHO 2008 report showed that Ethiopia ranks seventh among the world's 22 countries with high tuberculosis burden [5]. The Federal Ministry of Health (EFMOH) hospital statistics data showed that tuberculosis is the leading cause of morbidity, the third cause of hospital admission and the second cause of death in Ethiopia [6]. According to WHO 2007 report, the prevalence and mortality rate of all forms of TB in Ethiopia was estimated to be 546 and 73 per 100,000 populations respectively but based on WHO 2011 report the estimated prevalence and mortality rate was 394 and 35 per 100,000 respectively [1,7].

Tuberculosis can involve any organ system in the body. Although pulmonary tuberculosis is the most common presentation, extra pulmonary tuberculosis (EPTB) is also an important public health problem in this era of HIV/AIDS [8]. Review from Pittsburgh revealed that the increase in pathology associated with HIV/*M*. *tuberculosis* co-infection is caused by a functional disruption of the local immune response within the granuloma. These disruptions presumably decrease the ability of the granuloma to contain *M*. *tuberculosis*, leading to increased bacterial growth with more mycobacterial dissemination and severe pathology [9]. In developed countries, 10-15% of TB cases have extra-pulmonary involvement, but in patients from high-incidence countries the rate is much higher [10]. People who are HIV positive and infected with TB develop EPTB much more frequently, about 50% of cases [10]. In countries with comprehensive diagnostic and reporting systems, EPTB accounts for 20–25% of reported cases [11].

Extra pulmonary tuberculosis has the reverse epidemiological trend of pulmonary tuberculosis (PTB). Over the last several years, reported EPTB was increasing in absolute numbers and proportion of all reported TB cases [12]. Several studies confirmed that extra pulmonary TB is increasing from time to time. Retrospective study from Nigeria showed that, the proportion of EPTB notified remained consistently below 3% but during 2007 to 2009 it has risen to 5% [13]. Another study from Kenya demonstrated that EPTB rises from 6.4% to 16.7% between 1994 and 1997 [14]. In Ethiopia, according to WHO 2011 report, from the total of 156, 928 new TB cases notified in 2010, extra pulmonary TB accounts about 50,417 (32%) [1].

Specimens from extra-pulmonary sites chiefly consisted of body fluids, aspirates & biopsies from lymph nodes and other body sites. Of specific forms of EPTB, lymphadenitis, pleural, and bone/joint diseases are the most common [15]. Appropriate specimens might be difficult to obtain from extra-pulmonary sites and the number of bacilli is generally low. However, concentrating the sample (body fluids) with centrifugation may increase the chance of getting the bacilli in acid fast bacilli (AFB) stain. In Ethiopia, particularly in Gondar, little is known about the prevalence of smear positive extra pulmonary TB. Hence this study was designed to determine the magnitude of smear positive extra pulmonary tuberculosis infection among suspected patients at University of Gondar Hospital.

## Methods

### Study design and period

A cross sectional study was conducted from January 2012 to April 2012 to assess the prevalence of smear positive extra pulmonary tuberculosis infection among extra pulmonary tuberculosis suspected patients at University of Gondar Hospital.

### Study area

The study was conducted at University of Gondar Hospital, Gondar town, northwest Ethiopia. It is located 737 kilometers away from the capital city of Ethiopia, Addis Ababa in North Gondar zone of Amhara region. The town has latitude and longitude of 12^0^36^1^N and 37^0^28^1^E respectively with an elevation of 2133 meters above sea level. It has 13 urban and 11 rural kebeles with a projected population of 300,000 (Gondar Zonal statistics office). The hospital gives different inpatient and outpatient services to the population in the surrounding area of Gondar town and the adjacent regions.

### Source population and study population

The source population was all patients who were suspected for extra pulmonary tuberculosis infection and had access to visit University of Gondar Hospital whereas the study population was all extra pulmonary TB suspected patients who visited University of Gondar Hospital during the study period. Accordingly, a total of 344 study subjects were incorporated in this study.

### Operational definition

• Extra pulmonary tuberculosis suspected patients: Any person who presents with signs and symptoms of extra pulmonary tuberculosis including patients with chronic lymphadenitis clinically diagnosed as tuberculosis and body fluid accumulations with clinical suspicion of tuberculosis.

• Smear positive extra pulmonary tuberculosis: patient specimens such as FNA and body fluids that are positive for acid fast bacilli by Ziehl-Neelsen stain.

• Underlying diseases: Are diseases that compromise the immune system of persons or made their body weaker and more susceptible to infection or disease.

### Data collection methods

#### Administration of questionnaire

Pretested semi-structured questionnaire, which was first prepared in English and then translated into the local language (Amharic) during data collection, was used to collect socio-demographic characteristics of the patients and other explanatory variables. The data collectors were trained laboratory technologists from pathology department.

#### Sample collection

Samples from suspected patients for extra pulmonary TB were taken with fine needle aspiration (FNA) by a pathologist as routine procedure. On the other hand, the left over body fluids such as CSF, pleural fluid, peritoneal fluid and ascitic fluids that were sent to the clinical laboratory was also investigated. For all extra pulmonary tuberculosis positive patients the HIV status was determined by rapid HIV screening method at the Providing Initiative Testing and Counseling (PITC) clinic by nurses and the data was collected from the hospital record.

#### Human immunodeficiency virus test (HIV-test)

To detect HIV, the anti-HIV antibody test was used for screening according to the manufacturer’s instructions (rapid test currently used in national algorithm for Ethiopia). KHB (Shangha Kehua Bio-engineering Co., Ltd. China) was used for screening and positive samples were re-tested with STAT-PACK (Chembio HIV 1/2 STAT-PAK™ Assay, CHEMBIO DIAGNOSTIC SYSTEMS, INC., MEDFORD, NY, USA). Samples giving discordant results in the two tests were re-examined using tie-breaker, (Uni-Gold HIV, Trinity Biotech PLC, Co. Wicklow, Ireland).

#### Quality control

The questionnaire was pretested before the actual study began to make sure that whether the questionnaire was appropriate and understandable. The collected data was checked daily for consistency and accuracy. The appropriateness of the reagents was rechecked with a known positive and negative sample. Randomly positive and negative smears were blindedlly rechecked by an experienced pathologist and microbiologist for quality assurance and smears from known positive and negative specimens were used as positive and negative controls for internal quality control.

#### Specimen processing and laboratory investigation

After centrifugation of body fluid specimens at 3000xg for 15 minutes, supernatant was discarded and pellets were re-suspended with 500μl of phosphate buffer saline. Ten μl of the pellet was deposited on a clean microscope slide and spread over an area of approximately 2cm^2^ using the side of the pipette tip [16,17]. Smears were air dried and heat fixed. In the case of FNA samples, aspirated material was smeared on clean glass slides. The slides were air dried and fixed with heat. Slides were stained, with ZN method [18]. Finally the stained smears were examined under the oil immersion objective to look for acid fast bacilli in a light microscope (Olympus C × 31- Japan). A minimum of 100 oil immersion fields were observed to declare negative smear.

For cytological diagnosis, some of the slides were air dried and stained with Wright stain and examined by experienced pathologist. The evidences for diagnosis of tuberculosis by cytological examination of FNA samples were the presence of epitheloid granuloma with or without multinucleated giant cells and with or without caseus necrosis and / liquefied necrotic material with degenerating and viable inflammatory cells without epitheloid granuloma [19]. For pleural and peritoneal fluids exudative effusion with lymphocyte predominance was suggestive of tuberculosis.

Data entry and analysis were made using SPSS Version 16.0 statistical software. A bivariate analysis using binary logistic regression was done to determine the presence of a statistically significant association between explanatory variables and the outcome variables. To identify independently associated factors, multivariate logistic regression model was produced by taking presence of extra pulmonary tuberculosis as an outcome variable. All explanatory variables that were associated with the outcome variable in the bivariate analysis (P ≤ 0.2) and variables consistently found to be associated with occurrence of TB in other studies were included in the multivariate logistic regression model. Odds Ratio (OR), p-value and their 95% Confidence Intervals (CI) were calculated and the result was considered statistically significant at P < 0.05.

### Ethical considerations

The study was conducted after obtaining institutional ethical clearance from ethical review committee of School of Biomedical and Laboratory Sciences, University of Gondar. Informed consent was also obtained from the study participants. The result of the study participants who were positive for EPTB was reported to physicians for treatment. Information obtained at any course of the study was kept confidential. In addition, the clinical specimen collected during the study period was used for the stated objectives only and the study participants were participated once in the study period.

## Results

### Socio demographic characteristics of extra pulmonary tuberculosis suspected patients

A total of three hundred and forty four patients suspected of extra pulmonary tuberculosis infection were enrolled in this study. The socio-demographic characteristics of EPTB suspects showed that, there were 181 (52.6%) males and 163 (47.4%) females. The highest number of study participants was rural dwellers, 232 (67.4%). The mean age of the study subjects was 30.02 (SD. ±16.401). The majority of the study subjects were farmers 125 (36.3%) followed by housewife 75 (21.8%) and students 74 (21.5%). The educational status showed that 161 (46.8%) were illiterate, 104 (30.2%) completed primary school with the list of pre-school age, 20 (5.8%). Regarding to the marital status, there were 175 (50.9%) married, 126 (36.6%) single, 39 (11.3%) divorced and 4 (1.2%) widowed participants. Young adults age less than or equal to 30 years accounted more than half, 203 (59%) of the study subjects and especially the peak age group of 21–30 formed about one-third of the total extra pulmonary tuberculosis suspected patients, 94 (27.3%) (Table 1).

**Table 1:** **Socio-demographic characteristics and extra pulmonary tuberculosis among suspected patients at University of Gondar Hospital**, **from January 2012 to April 2012**, (**N** = **344**)

**Socio-demographic variables**	**Extra pulmonary TB**		**Total(%)**
	**Positive, No(%)**	**Negative, No(%)**	
**Sex**			
Female	14 (8.6)	149 (914)	163 (47.4)
Male	20 (11)	161 (89)	181 (52.6)
**Age**			
<10	3 (8.1)	34 (91.9)	37 (10.8)
10-20	7 (9.7)	65 (90.3)	72 (20.9)
21-30	8 (8.5)	86 (91.8)	94 (27.3)
31-40	10 (14.9)	57 (85.1)	67 (19.5)
41-50	3 (7.9)	35 (92.1)	38 (11.0)
>50	3 (8.3)	33 (91.7)	36 (10.5)
**Residence**			
Urban	14 (12.5)	98 (87.5)	112 (32.6)
Rural	20 (8.6)	212 (91.4)	232 (67.4)
**Occupation**			
Merchant	0 (0)	15 (100)	15 (4.4)
Student	9 (12.2)	65 (87.8)	74 (21.5)
Housewife	6 (8)	69 (92)	75 (21.8)
Daily-labor	3 (10)	27 (90)	30 (8.7)
Government employee	2 (12.5)	14 (87.5)	16 (4.7)
Farmer	13 (10.4)	112 (89.6)	125 (36.3)
Other	1 (11.1)	8 (88.9)	9 (2.6)
**Educational level**:			
Pre-school age	1 (5)	19 (95)	20 (5.8)
Not read and write	17 (10.6)	144 (89.4)	161 (46.8)
Primary school	9 (8.7)	95 (91.3)	104 (30.2)
Secondary school & above	7 (11.9)	52 (88.1)	59 (17.2)
**Marital status**:			
Single	15 (11.9)	111 (88.1)	126 (36.6)
Married	13 (7.4)	162 (92.6)	175 (50.9)
Divorce	6 (15.4)	33 (84.6)	39 (11.3)
Widowed	0 (0)	4 (100)	4 (1.2)
**Family size**			
≤4	20 (12.2)	14 (87.8)	164 (47.7)
≥5	14 (7.8)	166 (92.2)	180 (52.3)
**Number of house rooms**			
1	28 (9.9)	256 (90.1)	284 (82.6)
2	5 (10.9)	41 (89.1)	46 (13.4)
3	1 (7.1)	13 (92.9)	14 (4.1)

### Prevalence of extra pulmonary tuberculosis among suspected patients

Out of three hundred and forty four EPTB suspected clients 34 (9.9%) were AFB positive by Ziehl Neelsen staining technique. The prevalence of extra pulmonary tuberculosis among male and female cases were 20/181 (11.05%) and 14/163 (8.6%) respectively. From the positive cases the highest proportion (58.8%) was males. The uppermost prevalence of extra pulmonary TB cases was observed in the age group of 31–40, 10/67 (14.9%) and it was also highest among urban dwellers, 14/112 (12.4%) than rural, 20/232 (8.6%). In the case of marital status even though the majority of study subjects were married, the highest prevalence of extra pulmonary tuberculosis was seen among divorced patients, 6/39 (15.4%) (Table 1). The majority of EPTB cases were also observed among those whose average monthly income was less than 400 Ethiopian Birr, 13/63 (20.6%) (Table 2). All EPTB positive patients had weight loss, fever and loss of appetite. Moreover, 25 (73.5%), 33 (97.1%) and 32 (94.1%) had cough, night sweating and chest pain respectively.

**Table 2:** **Association of independent variables with the occurrence of EPTB at University of Gondar Hospital**, **from January 2012 to April 2012**, (**N** = **344**)

**Variables**	**EPTB status**		**COR (CI)**	**AOR (CI)**	**P-value**
	**Positive,No(%)**	**Negative,No(%)**			
**Residence**					0.346
Urban	14 (12.5)	98 (87.5)	1.51 (0.73-3.13) *	---------------	
Rural	20 (8.6)	212 (91.4)	1.00		
**Ingestion of raw milk**					0.162
Yes	28 (15.1)	158 (84.9)	4.49 (1.81-11.15) * *	--------------	
No	6 (3.8)	152 (96.2)	1.00		
**Monthly income**					
<400	13 (20.6)	50 (79.4)	13.78 (2.99-63.4)* *	12.9 (2.25-68.02)	0.018
400-550	14 (13.3)	91 (86.7)	8.15 (1.81-36.83)	9.77 (1.92-49.68) 5.1	
551-800	5 (7.4)	63 (92.6)	4.21 (0.79-22.33)	(0.23-31.48) 1.00	
>800	2 (1.9)	106 (98.1)	1.00		
**Family size**				----------------	0.270
≤4	20 (12.2)	14 (87.8)	1.00		
≥5	14 (7.8)	166 (92.2)	0.61 (0.29-1.25) *		
**Number of house of rooms**					
**1**	28 (9.9)	256 (90.1)	1.42 (.179-11.28) *	----------------	0.106
**2**	5 (10.9)	41 (89.1)	1.59 (.169-14.83)		
**3**	1 (7.1)	13 (92.9)	1.00		
**TB contact**					<0.001
Yes	19 (26.8)	52 (73.2)	6.29 (3.0-13.17)* * *	6.67 (2.78-16.90)	
No	15 (5.5)	258 (94.5)	1.00	1.00	
**History of TB**					
Yes	16 (34)	31 (66)	8.0 (3.71-17.26)* * *	4.77 (1.86-12.24)	0.001
No	18 (6.1)	279 (93.9)	1.00	1.00	
**Underlined Diseases**					
Yes	23 (19.8)	93 (80.2)	4.88 (2.3-10.4)* * *	2.79 (1.15-6.78)	0.023
No	11 (4.8)	217 (95.2)	1.00	1.00	
**Specimen**					
FNAC	28 (12.7)	193 (87.3)	2.40 (1.02-5.69) * *	2.94 (1.07-8.09)	0.036
Body fluid	6 (4.9)	117 (95.1)	1.00	1.00	

**Key**: *** P-value <0.001, ** p-value < 0.05, * p-value >0.05.

### Extra pulmonary tuberculosis prevalence at different body sites

**Figure 1:**
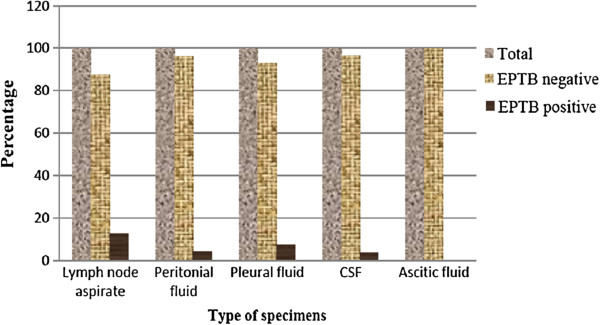
**Smear positive extra pulmonary tuberculosis prevalence among different extra pulmonary specimens at University of Gondar Hospital**, **from January to April 2012.**

Lymph node aspirates, peritoneal fluid, pleural fluid, cerebrospinal fluids and ascitic fluids were examined for extra pulmonary tuberculosis infection. Lymph node aspirate was the highest in number, 221 (64.2%) of specimens involved followed by peritoneal fluid, 49 (14.2%) and pleural fluid 43 (12.5%). The prevalence of smear positive extra pulmonary tuberculosis infection among lymph node aspirate and body fluids were 28/221 (12.7%) and 6/123 (4.9%) respectively (Figure 1). Of the smear positive extra pulmonary tuberculosis cases, lymph node tuberculosis infection was constituted the largest proportion, 28/34 (82.4%) followed by pleural fluids, 3/34 (8.8%).

### The HIV status of smear positive extra pulmonary tuberculosis patients

**Figure 2:**
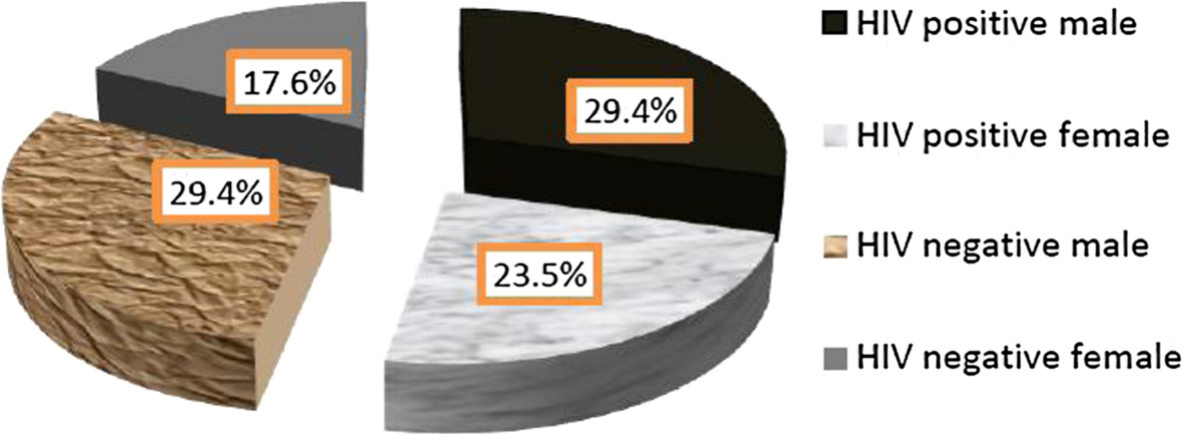
**The proportion of HIV infection among smear positive extra pulmonary tuberculosis patients at University of Gondar Hospital**, **from January to April 2012.**

Out of 34 EPTB positive patients by Ziehl Neelsen staining technique, more than half of them, 18 (52.9%) were also positive for HIV. Among extra pulmonary tuberculosis and HIV co-infected patients, the highest cases were TB lymphadenitis, 12 (66.7%). The proportion of HIV infection was almost equally distributed between male and female patients with slightly higher in males than females (29.4% versus 23.5%) (Figure 2). The highest proportion of HIV infection was observed in the age group of 31–40, (9(50%)).

### Cytology result of extra pulmonary tuberculosis infection

From a total of two hundred and forty four EPTB suspected patients who were diagnosed cytologically, 206 (84.4%) were from lymph node aspirate and more than half of them, 140 (63.9%) were diagnosed as extra pulmonary tuberculosis positive. Moreover, 23 (16.4%) of these patients were smear positive by Ziehl Neelsen staining technique. On the other hand, among 37 (15.6%) body fluids samples, 12 (31.6%) were cytologically suggestive of extra pulmonary tuberculosis infection but only one was confirmed as smear positive with Ziehl Neelsen staining technique. The overall diagnosis result of EPTB with cytological investigation was 152 (62.3%) (Table 3).

**Table 3:** **Distributions of EPTB with in Age group by AFB microscopic and cytological techniques among suspected patients at University of Gondar Hospital**, **from January 2012 to April 2012**, (**N** = **344**)

**Age**	**Sex**		**Types of specimens**					**AFB microscopic results**		**Cytological results**	
	**Male**	**Female**	**Lymph node (%)**	**CSF (%)**	**Periton-eal fluid (%)**	**Pleural Fluid (%)**	**Ascitic fluid (%)**	**Positive (%)**	**Negative (%)**	**Positive (%)**	**Negative (%)**
<10	17	20	24 (10.9)	1 (2)	3 (7)	9 (33.3)	0 (0)	3 (8.1)	34 (91.9)	13 (59)	9 (41)
10-20	34	38	51 (23.1)	7 (14.3)	6 (14)	8 (29.6)	0 (0)	7 (9.7)	65 (90.3)	33 (60)	22 (40)
21-30	52	42	69 (31.2)	10 (20.4)	11 (25.6)	3 (11.1)	1 (25)	8 (8.5)	86 (91.5)	33 (62.3)	20 (37.7)
31-40	27	40	43 (19.5)	13 (26.5)	6 (14)	3 (11.1)	2 (50)	10 (14.9)	57 (85.1)	46 (67)	23 (33)
41-50	17	21	20 (9)	9 (18.4)	8 (18.6)	1 (3.7)	0 (0)	3 (7.9)	35 (92.1 )	16 (61.5)	10 (38.5)
>50	16	20	14 (6.3)	9 (18.4)	9 (21.1)	3 (11.1)	1 (25)	3 (8.3)	33 (91.7)	11 (57.9)	8 (42.1)
Total	163	181	221 (100)	49 (100)	43 (100)	27 (100)	4 (100)	34 (9.9)	310 (90.1)	152 (62.3)	92 (37.7)

### Socio-demographic characteristics and possible associated risk factors among extra pulmonary tuberculosis suspected patients

Multivariate and bivariate logistic regression analysis was conducted to evaluate the significance of association between EPTB and explanatory variables. Socio demographic variables such as age, sex, educational status, residence and occupational status of the respondents were not significantly associated with extra pulmonary tuberculosis infection in this study. Individuals who had previous history of underlining diseases were about 3 times more likely to have EPTB than those who had not (AOR = 2.79, 95% CI, 1.14-6.78). The diagnosis of tuberculosis by cytology as well as AFB stain was higher in lymph node samples compared to other body fluids (AOR = 2.945, 95% CI, 1.073-8.086). Extra pulmonary tuberculosis patients whose average monthly income less than 400 Ethiopian birr were about more than ten times more likely to have had EPTB than those whose income was greater than 800 Ethiopian birr (AOR = 13.78, 95% CI 2.99-63.4). Extra pulmonary tuberculosis infection suspected patients who had previous history of TB were about 5 times more likely to have EPTB than those who did not have previous history of TB, (AOR = 4.77, 95% CI 2.509-35.527). Patients who had history of tuberculosis contact to a known TB cases were also about 6 times more likely to have risk of EPTB than those who had not TB contact (AOR = 6.67, 95% CI, 2.636-29.326). History of ingestion of raw milk was higher, 28/34 (82.4%), among EPTB positive patients and it was statistically significant with bivariate analysis (COR = 4.49, 95% CI 1.81-11.15).

## Discussion

Extra pulmonary tuberculosis is one of the highly prevalent diseases in developing countries including Ethiopia. In this study the prevalence of smear-positive extra pulmonary tuberculosis infection was 9.9% which is higher than the prevalence reported in Nigeria (5%) and India (3.9%) [13,20] but lower than the prevalence observed in Turkey (25.9%), Southern region of Ethiopia (28%) and Addis Ababa (15.9%) [21-23]. previously, the prevalence of extra pulmonary tuberculosis infection was reported 28.3% in Gondar [24]. The discrepancy between the current study and previous reports on EPTB infection might be the result of variation in diagnostic methods; the majority of the previous reports were done by culture, cytological method and/or PCR but smear microscopy was the only procedure used in the current study. Nevertheless, extra pulmonary tuberculosis remains a disease of major public health importance and it has been increasing from time to time. The possible reasons why EPTB infection is rising might be possibly due to; high tuberculosis burden, increase in HIV pandemic, under diagnosis of pulmonary tuberculosis or transmission of other mycobacterium such as *M*. *bovis*[25].

Among two hundred and forty four EPTB suspected patients who were diagnosed using cytological method, the overall magnitude of EPTB infection was 152/244 (62.3%). From those, one hundred and forty specimens were lymph node aspirates and 12 other body fluids. Twenty-three lymph node aspirates and one body fluid specimens were also smear positive with Ziehl Neelsen staining technique. The cytological result of the current study was higher than the other studies conducted in Saudi Arabia (42.9%) and Ethiopia (39.7%) [26,27].

In the current study, the socio-demographic characteristics like sex, age, marital status, occupation and educational status were not significantly associated with extra pulmonary tuberculosis infection. This might be due to the small sample size. However, although marital status was not significantly associated with the EPTB infection, about half of the study subjects were married but the highest prevalence of EPTB cases was observed among divorced individuals, 6/39 (15.4%). Most of HIV positive patients in this study were also among divorced persons and then this could be one possible reason for the highest prevalence of EPTB among this group of individuals. Male patients were more likely to present with extra pulmonary tuberculosis than females (58.8% versus 41.2%). This is supported by other reports from India, (65.6% versus 34.4%), Malatya/Turkey, (53.4% versus 46.6%) and Nigeria, (64.6 % versus 35.4%) [21,28,29]. However, there are contradictory reports on the EPTB prevalence among male and female patients. Adriano et al. and others reported that women were more likely to have EPTB than men in California, USA and Nepal [30-32].

The prevalence of extra pulmonary tuberculosis infection among patients with age group 31–40 years was relatively higher with both direct AFB microscopic (14.9%) and cytological 46 (67%) techniques in the current study (Table 3). Previously, age was reported as an independent risk factor for EPTB infection in high-burden countries [26,32,33]. This is consistent with other studies in San Francisco, Saudi Arabia, India and Tanzania which showed that high prevalence of EPTB among productive age groups [26,28,30,34]. Most of tuberculosis cases in this population might be largely related to ongoing tuberculosis transmission, given that the associated age group (31–40 years) is known as the most unstable and high risk group for TB transmission. In the case of this study the rate of HIV infection was higher in this age group and this might be one possible reason. This leads to grave socio-economic consequences in a country with a very high prevalence of the disease.

Previous history of tuberculosis infection and contact to a known tuberculosis cases were significantly associated with extra pulmonary tuberculosis infection in the current study. This is supported by other studies conducted in Pakistan and Ethiopia which documented that the risk of developing EPTB infection among patients with previous history of tuberculosis infection and TB contact cases [23,35]. More over patients who had history of underlying disease other than TB were also prone to have EPTB than those who had not. This could be due to an immune suppression of individuals with those diseases which makes them being susceptible for tuberculosis infection.

In this study, income was found as one of the risk factors for EPTB infection. Tuberculosis spreads easily in overcrowded, badly ventilated places and among people who are undernourished; as tuberculosis is known as a disease of poverty [36].

A significant number of EPTB cases had history of ingestion of raw milk, 28/34 (82.4%) in this study. Ingestion of raw milk is one of the risk factor for extra pulmonary tuberculosis infections. *Mycobacterium bovis* was previously reported as an ethological agent for EPTB infection as a result of zoonotic transmission mainly through milk ingestion. The study from pastoralist of Somali Regional State, Ethiopia showed that consumption of raw milk and sharing same accommodation (same fence) with livestock were found highly prevalent [37]. However, recent study reported that no *M*.* bovis* isolated from TB lymphadenitis patients in Ethiopia (unpublished source [38]).

Among 34 EPTB confirmed patients over half of them were also infected with HIV, 18 (52.9%). Similar findings had been observed in Tanzania (58.3%) and Ethiopia (50.6%) [23,34]. The distribution of HIV by sex was almost equal and it was highly prevalent in the age group of 31–40, (50%). Tuberculosis and HIV/AIDS are the two most common infectious diseases in developing countries and they have a synergetic interaction; each propagates progression of the other. Studies have shown that the magnitude of extra-pulmonary tuberculosis is increasing due to HIV infection [30,39]. Immune deficiency syndrome caused by HIV virus facilitates dissemination of tuberculosis infection from pulmonary tuberculosis cases as there is low or no granuloma formation during HIV-TB co-infection and functional disruption of the local immune response within the granuloma [9].

In this study tuberculosis lymphadenitis was found to be the commonest form of extra pulmonary tuberculosis, comprising 82.4% of the extra pulmonary tuberculosis cases. This finding is consistent with studies conducted in Rajshahi/Bangladesh (75%) and Ethiopia (67%) [27,40]. However, Kamenju et al. from Tanzania reported that most common sites of occurrence for EPTB were the pleural fluid, (44%) [34]. Furthermore, the prevalence of EPTB from lymph node aspirates and other body fluids were 12.7% and 4.9% respectively. The current study result of lymph node tuberculosis (12.7%) was slightly higher than the study conducted from Malawi (8%) [41].

The limitation of the present study was that only Ziehel Neelsen staining technique was used. The very low AFB score among extra pulmonary tuberculosis specimens which could result AFB negative as Ziehel Neelsen staining technique had low sensitivity.

## Conclusion and recommendation

Extra pulmonary tuberculosis remains a significant fraction of the total TB cases in developing countries. It is a diagnostic challenge in sub Saharan Africa, including Ethiopia, where there is a high rate of HIV infection. The frequency of smear positive EPTB in this study was high with the highest proportion of lymph node aspirate. Moreover, being male patient was at higher rate of positivity for EPTB than female. Among EPTB confirmed patients, above half of the cases were HIV/AIDS positive and the highest frequency was among the productive age group which was coincided to the highest TB prevalence. Low income, previous history of TB, history of tuberculosis contact to a known TB cases and history of underlying diseases were significant likelihood factors for patient being EPTB positive. Based on the above conclusion the following recommendations are forwarded: Attention should be given to strengthen the laboratory services for EPTB diagnosis on investigating lymph node and other body fluids for tubercle bacilli in University of Gondar hospital in particular and in the country in general. Since contact with known tuberculosis cases is a significant factor, health education about tuberculosis transmissions and prevention should be given and large scale study on the prevalence of EPTB infection using culture or other more sensitive methods could maximize and determine the exact prevalence of EPTB infection.

## Competing interests

There was not any conflict of interest among the authors and with the others too.

## Authors’ contributions

YZ: Conceived, designed and proposed the research idea. YZ, WT, BG and BA: involved in data collection. YZ, BG, BA, WT, TD and FB: involved in data entry, clearance, analysis, and interpretation of the findings. YZ: responsible for drafting the manuscript. All authors involved in reviewing the manuscript and approval for publication.
